# Respiratory Syncytial Virus and Cellular Stress Responses: Impact on Replication and Physiopathology

**DOI:** 10.3390/v8050124

**Published:** 2016-05-12

**Authors:** Sandra L. Cervantes-Ortiz, Natalia Zamorano Cuervo, Nathalie Grandvaux

**Affiliations:** 1 CRCHUM—Centre Hospitalier de l’Université de Montréal, Montréal, QC H2X 0A9, Canada; s.l.cervantes.ortiz@umontreal.ca (S.L.C.-O.); Natalia.zamorano@umontreal.ca (N.Z.C.); 2 Faculty of Medicine, Université de Montréal, Montréal, QC H3C 3J7, Canada; 3 Department of Microbiology, Infectiology and Immunology, Université de Montréal, Montréal, QC H3C 3J7, Canada; 4 Department of Biochemistry and Molecular Medicine, Université de Montréal, Montréal, QC H3C 3J7, Canada

**Keywords:** virus, respiratory syncytial virus, RSV, stress response, endoplasmic reticulum, ER stress, stress granule, reactive oxygen species, oxidative stress, inclusion bodies

## Abstract

Human respiratory syncytial virus (RSV), a member of the *Paramyxoviridae* family, is a major cause of severe acute lower respiratory tract infection in infants, elderly and immunocompromised adults. Despite decades of research, a complete integrated picture of RSV-host interaction is still missing. Several cellular responses to stress are involved in the host-response to many virus infections. The endoplasmic reticulum stress induced by altered endoplasmic reticulum (ER) function leads to activation of the unfolded-protein response (UPR) to restore homeostasis. Formation of cytoplasmic stress granules containing translationally stalled mRNAs is a means to control protein translation. Production of reactive oxygen species is balanced by an antioxidant response to prevent oxidative stress and the resulting damages. In recent years, ongoing research has started to unveil specific regulatory interactions of RSV with these host cellular stress responses. Here, we discuss the latest findings regarding the mechanisms evolved by RSV to induce, subvert or manipulate the ER stress, the stress granule and oxidative stress responses. We summarize the evidence linking these stress responses with the regulation of RSV replication and the associated pathogenesis.

## 1. Introduction

Members of the *Paramyxoviridae* family include several etiological agents associated with a high burden of morbidity and mortality in humans. Amongst the *Paramyxovirinae* subfamily are the parainfluenza viruses (PIVs), measles virus (MV), mumps virus (MuV), Nipah virus (NiV) and Hendra virus (HeV), while the *Pneumovirinae* subfamily includes respiratory syncytial virus (RSV) and metapneumovirus (MPV) [1,2]. Human RSV is a major cause of acute lower respiratory tract infection in infants, children and elderly, but also all-aged adults with a compromised immune system or cardiopulmonary diseases. RSV-associated pathologies range from mild upper respiratory tract illness, including otitis and rhinitis, to the life-threatening acute lower respiratory tract diseases bronchiolitis and pneumonia. RSV infection at a young age is also associated with increased susceptibility to asthma development [3]. RSV significantly disrupts the airway and alveolar epithelium, induces airway plugging due to submucosal oedema and loss of cilia on the apical surface of infected epithelial cells combined with accumulation of mucus and cellular debris, and promotes neutrophil infiltration in the airways [4]. The severity of the clinical manifestations positively correlates with the virus titer and the exacerbated proinflammatory cytokine/chemokine response skewed toward a T helper type 2 (Th2) immune response, but the relative contributions of these parameters to the disease remain unknown [3]. Current treatment is limited to supportive care. Development of a RSV vaccine remains a challenge, but immunoprophylaxis with monoclonal anti-RSV fusion protein (Palivizumab) effectively confers protection. However, due to high costs, the prophylactic treatment is limited to children considered at high risk of developing severe disease, *i.e.*, preterm babies or infants with congenital heart disease [5].

Paramyxoviruses are enveloped viruses containing non-segmented negative-sense RNA genome. The 15.2 kb RSV genome is composed of 10 genes arranged sequentially on the template that encodes for 11 proteins. The large polymerase protein (L), the phosphoprotein (P) and the nucleoprotein (N) form the helical ribonucleoprotein complex which encapsidates the genomic RNA. The nucleocapsid is surrounded by the matrix (M) protein and an envelope derived from the host membrane in which the viral attachment glycoprotein (G), fusion glycoprotein (F) and small hydrophobic glycoprotein (SH) are anchored. The SH glycoprotein is specific to RSV amongst *Paramyxoviridae*. Other additional genes encoded by RSV that are not present in other paramyxoviruses include the M2-1 and M2-2 involved in transcription and replication, respectively, and two non-structural proteins, NS1 and NS2 [3,6].

RSV primarily targets ciliated airway epithelial cells (AECs), but immune cells such as dendritic cells (DCs) and macrophages can also be infected with RSV [7,8,9,10]. RSV replication cycle generally follows the general scheme of the paramyxoviruses [11,12]. Nucleolin expressed at the surface of AEC has recently been proposed as a functional receptor for RSV that binds the RSV F protein and is required for RSV fusion with the plasma membrane [13]. Fusion occurs after a transient macropinosome formation to deliver the nucleocapsid in the cytoplasm [14], where transcription and replication occur. Transcription and replication are regulated by a unique promoter in the 3′ leader (Le) region of the genome. Transcription by the RNA-dependent-RNA-polymerase composed of L and P proceeds directly from the negative sense (3′-5′) genome through the production of capped/polyA monocistronic mRNAs. Replication involves the formation of an antigenome (5′-3′) intermediate. The 5′ end of the genome contains an extragenic trailer sequence (Tr). The complement of the Tr sequence (TrC) found at the 3′ end of the antigenome contains a promoter required for the synthesis of new genomic RNA [3,6]. Transcription and replication of RSV genome generate specific RNA intermediates that constitute pathogen-associated molecular patterns (PAMPs), which are sensed by pattern recognition receptors (PRRs) to trigger the interferon (IFN)-mediated antiviral response and the expression of proinflammatory cytokines. RSV exhibits a complex regulatory interaction with different PRRs. RSV sensing in the cytoplasm involves the retinoic acid-inducible gene I (RIG-I)-like receptors (RLRs)—RIG-I and melanoma differentiation-associated protein 5 (MDA5)—and the double-stranded RNA (dsRNA)-dependent protein kinase R (PKR). Toll-like receptors (TLRs) 2, 3, 4 and 7 localized in the membrane or in the endosomal compartment also contribute to RSV sensing in a cell-type-specific manner [15].

Many viruses, including paramyxoviruses, have evolved mechanisms that permit selective translation of their mRNAs under shutoff conditions imposed by the IFN and cellular stress responses [16]. However, RSV counts amongst the viruses that replicate efficiently without inducing an overall inhibition of host cellular protein synthesis [17]. As numerous viruses, RSV has evolved mechanisms to hijack the IFN antiviral response, mainly through the NS1 and NS2 proteins. These mechanisms have been recently reviewed [18]. Here, we focus on the specific regulatory interactions of RSV with the host cell stress responses, the endoplasmic reticulum (ER) stress, the stress granule (SG) response, and oxidative stress to illustrate their relationship with RSV viral replication and physiopathology.

## 2. RSV Induces ER Stress and a Non-Canonical Unfolded-Protein Response (UPR)

The ER is a cellular organelle essential for the control of several cellular functions including nascent protein folding and post-translational modifications [19]. Upon dysfunction of the ER, known as ER stress, the cell engages in a complex evolutionary conserved process, the UPR response, to restore homeostasis. Initiation of the UPR response relies on the activation of three transmembrane ER stress sensors: activated transcription factor 6 (ATF6), PKR-like ER kinase (PERK) and inositol-requiring enzyme 1 (IRE1). Each of these branches of the UPR response contributes to the reduction of protein translation, while promoting the protein-folding capacity and degradation of unfolded proteins through the induction of ER chaperones, “ER-associated protein degradation” (ERAD) proteins, and autophagy to decrease ER protein-folding load. If homeostasis is not recovered, the UPR response switches from being pro-survival to being pro-apoptotic [20,21]. ER stress is induced by numerous viruses either as a result of ER membrane exploitation, misfolded-protein accumulation, imbalance of calcium concentration or sabotage/depletion of the ER membrane during virion release [22]. Accordingly, manipulation of the UPR response has become an asset for many viruses to promote their translation [21,23].

**Figure 1:**
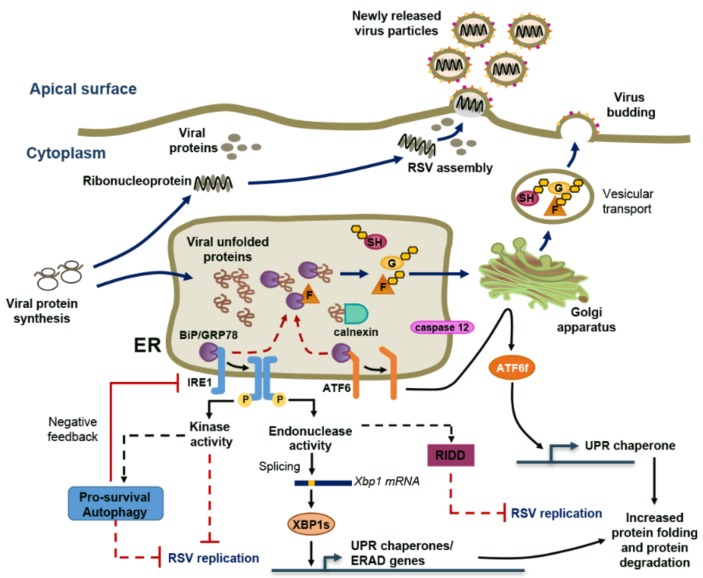
RSV Induces a Non-Canonical UPR Response. Endoplasmic Reticulum (ER) stress induction upon Respiratory Syncytial Virus (RSV) infection, possibly as a result of ER overload due to glycoprotein RSV F, G and SH transit, is associated with activation of the inositol-requiring enzyme 1 (IRE1) and activated transcription factor 6 (ATF6) branches of the unfolded protein response (UPR). Interaction of F with the binding immunoglobulin protein (BiP/GRP78) chaperone could be an initiating event of the UPR response. Increased protein folding and degradation capacity are induced as a result of X-box binding protein 1 (XBP1) splicing variant XBP1s production by IRE1 endonuclease activity and ATF6f fragment formation. IRE1 acts as a restriction factor against RSV independently of XBP1s. IRE1 kinase activity and IRE1-endonuclease activity involved in selective mRNA degradation, a process called IRE1-dependent decay (RIDD), might contribute to IRE1-dependent inhibition of RSV replication through mechanisms that remain to be characterized. Autophagy was shown to promote negative feedback on the IRE1 pathway.

RSV-mediated activation of ER stress has been shown in primary human tracheobronchial epithelial (HTBE) cells and in the A549 cell line. The response is associated with an incremental induction of the ER chaperones, binding immunoglobulin protein (BiP, also known as GRP78) and calnexin, and of caspase 12, which are typically upregulated in response to high ER unfolded-protein load [24,25]. The exact mechanism triggering ER stress during RSV infection remains elusive. Positive-strand RNA viruses, such as hepatitis C or dengue virus, exploit the ER membranes to form a platform for their replication, thereby causing ER stress [26,27]. However, there is no such report for RSV or any other paramyxoviruses which replicate in cytoplasmic SGs/inclusion bodies (IBs) as discussed below [28,29,30,31]. A possible mechanism for ER stress induction during RSV infection involves the production of large amounts of glycoproteins, which likely compete with host proteins for processing and induce an increased burden on ER/Golgi trafficking [22,32]. *De novo* production and assembly of RSV virions highly rely on the transit of the three glycosylated surface proteins, the heavily glycosylated G protein and the F and SH proteins, through the ER, the Golgi apparatus and the secretory pathway to reach the apical membrane where they will assemble with the M, N, P, M2-1, M2-2 proteins and RSV RNA contained in cytoplasmic IBs [33,34,35,36,37] (Figure 1). Similar to what is observed with the hemagglutinin-neuraminidase (HN) glycoprotein of hPIV5 (also known as simian virus 5), the RSV F protein interacts with the BiP/GRP78 ER chaperone during the transit [38,39]. An early event required for the activation of the UPR response is the dissociation of ATF6/IRE1/PERK from the BiP/GPR78 [20,21]. Although it has yet to be demonstrated that the RSV F/GPR78 interaction results in competitive dissociation from ATF6/IRE1/PERK, this is a likely mechanism that might at least in part contribute to RSV-induced UPR response (Figure 1). Virus-encoded viroporins form aqueous channels modifying host cellular membrane permeability to ions and small molecules. Viroporins alter ER function in infected cells through the induction of ER Ca^2+^ leakage, ER membrane rearrangement or modulation of intracellular glycoprotein trafficking, and are increasingly found implicated in the modulation of the UPR response [40]. This has been well illustrated in the context of severe acute respiratory syndrome coronavirus (SARS-CoV) infection. The SARS-CoV E and 3a proteins, both of which exhibit viroporin functions, have opposite effects on ER stress. SARS-CoV E inhibits IRE1-dependent UPR response, including in the context of RSV infection, while SARS-CoV 3a protein induces the PERK branch [41,42]. The SH glycoprotein of RSV acts as a viroporin at the plasma membrane and in the Golgi apparatus where it accumulates in lipid raft structures and forms ion channels selective of Na+ and K+ [43,44]. Not much is known about the function of RSV SH except that it contributes to the activation of the nucleotide-binding domain and leucine-rich repeat, pyrin domain containing 3 (NLRP3) inflammasome and to the inhibition of apoptosis, two mechanisms that, as described above, are regulated by ER stress [44,45]. This suggests a potential mechanistic link between RSV SH and the regulation of ER stress that would deserve further investigation.

Analysis of the activation of individual branches of the UPR response following RSV infection revealed an induction of ATF6-dependent promoter activity and IRE1-dependent X-box binding protein 1 (XBP1) splicing [25]. The lack of the concomitant detection of PERK phosphorylation, used as a read-out of its activation, led to the conclusion that RSV induces a non-canonical UPR response [25] (Figure 1). Selective activation of UPR branches is not specific to RSV, but has also been observed in response to other viruses, such as tick-borne-encephalitis, which, similar to RSV, activates ATF6 and IRE1, or influenza A, which selectively activates IRE1. In these infections, activation of specific UPR branches appears to give an advantage to the virus favoring its replication [46,47]. In contrast, inhibition of IRE1 endonuclease activity in A549 cells, using the 3,5-dibromosalicylaldehide (DBSA) inhibitor, or infection of IRE1 knockout (KO) mouse embryonic fibroblasts (MEFs) results in increased RSV mRNA and protein levels and *de novo* production of infectious virions compared to control cells [25]. Thus, IRE1 appears to act as a restriction factor during RSV infection [25]. Whether this is associated with the role of IRE1 in the splicing of XBP1, which generates the transcriptionally active form of XBP1—XBP1s—remains unclear. Indeed, RSV replication is not affected when performed in XBP1 KO MEFs compared to wild-type (wt) MEFs [25]. Alternatively, IRE1-dependent RSV mRNA decay might result from the nuclease activity of IRE1 that promotes the degradation of specific mRNAs to alleviate the rate of mRNA translation, a process known as IRE1-dependent decay (RIDD) [48] (Figure 1). Additional functions of IRE1 involve its kinase activity, which is known to trigger a TRAF2/ASK1/MAPK signaling cascade ultimately modulating pro-survival, pro-apoptotic and autophagic responses and thereby could contribute to the IRE1-dependent, XBP1s-independent restriction of RSV [49]. IRE1 is generally known to induce pro-survival autophagy through mechanisms involving XBP1 splicing and c-Jun N-terminal kinase (JNK) activation. However, during RSV infection, the IRE1 branch exhibits intricate regulatory interactions with autophagy. Indeed, RSV infection of AEC derived from mice deficient in the critical autophagy protein LC3b (LC3b KO) leads to increased IRE1-dependent XBP1 splicing and target gene expression associated with induction of IL-6 and inflammasome-dependent production of IL-1β [50]. This suggests that autophagy contributes to a negative feedback loop to lessen the RSV-induced ER stress response (Figure 1). Overall, the description of the ER stress responses during RSV infection is still sparse. However, the IRE1-dependent UPR branch emerges as a cell response that antagonizes RSV replication through mechanisms that need further investigation. Additionally, the final outcome of the ATF6 pathway during RSV infection remains to be elucidated.

## 3. RSV Takes Advantage of Cytoplasmic SGs and Specific IBs

**Figure 2:**
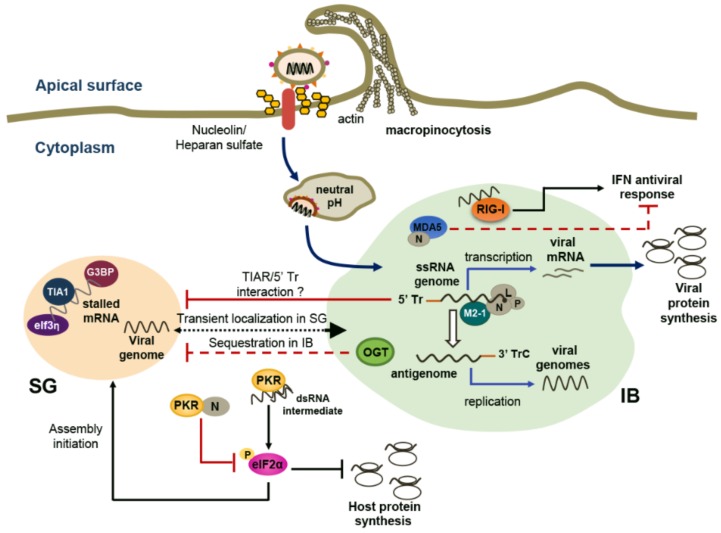
Crosstalk between SGs and IBs during RSV infection. Respiratory syncytial Virus (RSV) infection triggers the formation of cytoplasmic G3BP1/TIA1/eIf3η positive stress granules (SGs) and inclusion bodies (IBs). Viral genomic ssRNA transiently localizes within SGs, but is mainly associated in IBs. Colocalization of RSV ribonucleoprotein complex, composed of N, P and L, and of the M2-1 protein in IBs suggests that IBs favor RSV replication and transcription. IBs also contain the pattern recognition receptors, retinoic acid-inducible gene I (RIG-I) and melanoma differentiation-associated protein 5 (MDA5), which sense viral replication intermediates to trigger the interferon (IFN) antiviral response. Interaction of N with MDA5 is thought to counteract the antiviral response. Formation of SGs containing stalled mRNA to inhibit their translation is initiated upon activation of dsRNA-dependent protein kinase R (PKR) by dsRNA intermediates and downstream eIF2α phosphorylation. However, several mechanisms limit the formation of SGs. First, PKR-dependent eIF2α phosphorylation is limited by the interaction of RSV N with PKR. Second, the 5’ extragenic trailer sequence (5’ Tr) of the RSV genome interferes with SG assembly possibly through interaction with TIA1-related (TIAR) protein, as it was shown during SeV infection. Finally, RSV induces the redistribution of the O-linked *N*-acetylglucosamine transferase (OGT), an enzyme critical for assembly of stalled RNA into SGs, into IBs.

Amongst the plethora of responses to stress, the formation of RNA- and RNA-binding proteins (RNPs)-containing cytoplasmic foci, identified as SGs, provides a means for cells to sequester stalled mRNA and inhibit their translation [51]. The capacity of RNA viruses to induce, prevent or manipulate the formation of SGs varies according to viral species [52,53]. Amongst paramyxoviruses, formation of SGs has been evaluated in Sendai virus (SeV), MV and RSV infections [31,54,55]. In contrast to MV and SeV, which do not induce the formation of SGs, RSV triggers a persistent SG response characterized by the formation of RasGAP SH3 domain-binding protein 1 (G3BP1)/T-cell intracellular antigen (TIA1)/eukaryotic translation initiation factor 3η (eIf3η) positive granules [31,56,57] (Figure 2). However, SGs were consistently detected in a maximum of 30% of RSV-infected Hep-2 or A549 cells up to 24 h post-infection [31,56,57,58].

PKR-dependent eIF2α phosphorylation, a very early step associated with initiation of SG assembly, is observed during RSV infection; PKR knockdown experiments in Hep-2 cells have provided evidence that PKR is required for RSV-induced SG formation [56]. Although wtRSV induces activation of PKR, eIF2α phosphorylation is limited by the interaction of RSV N protein with PKR, suggesting that the N protein limits the initiation of SG formation [56,59] (Figure 2). An additional report has shown that the 5’ Tr sequence of the RSV genome, essential for optimal genome replication, also interferes with the propensity of RSV to elicit SG assembly. Infection with recombinant RSV lacking most of the Tr sequence induces increased SG response compared to wtRSV [57]. The mechanism by which the Tr region of the RSV genome impacts the SG response remains unknown. By analogy with the observation made in the context of SeV infection, a possible mechanism is that the Tr region binds to the SG component TIAR (TIA1-related) protein that is involved in SG assembly [54] (Figure 2). The sequestration of TIAR as a way of viruses to shut-off SG formation was also reported in the context of infection by the *Flaviviridae* West Nile virus and dengue virus [60]. This model is challenged in the context of RSV by the detection of SGs in TIAR KO MEFs infected with RSV containing a mutant Tr sequence [57]. However, a thorough quantification of RSV-induced SG formation in the absence of TIAR is required to conclude appropriately on TIAR/5′ Tr interaction as an interference mechanism. The incapacity of RSV to fully inhibit SG formation, in contrast to MV and SeV, might reflect the lack of expression of the C protein from the RSV genome. In the context of SeV or MV infection, the C protein that results from the translation from an alternative overlapping open reading frame of the *P* gene [12], is mainly responsible for interference with SG formation [55,61,62]. In contrast to wt viruses, MV or SeV mutants deleted for the C protein expression are strong inducers of G3BP1-positive SG formation. This observation correlates with the recognized role of the C protein to inhibit PKR-dependent eIF2α phosphorylation [55,61,63].

The formation of SGs was proposed to be necessary for efficient RSV replication based on the observation that interference with the expression of G3BP1, a protein essential for SG assembly, strongly reduced RSV titer and RSV RNA levels [31]. Although this suggests that RSV has evolved mechanisms to subvert SGs to its own advantage, this model was challenged by the lack of impact of the inhibition of SG formation through PKR silencing on RSV replication in Hep-2 cells [56]. At this point it cannot be excluded that G3BP is involved in RSV replication through a mechanism that is independent of SGs.

An additional report suggests that SGs are not the main sites of RSV replication. Indeed, RSV genomic RNA only transiently colocalizes with RSV-induced or arsenite-induced SGs [64] (Figure 2). Rather, viral genomic RNA predominantly colocalizes with cytoplasmic IBs that are distinct from SGs [31]. IBs are sites of N, P, L, M2-1 accumulation, which is consistent with a role in the viral ribonucleocapsid assembly and viral RNA synthesis and replication [28,58,65]. Interestingly, occurrence of cytoplasmic IBs observed at an early stage of RSV infection seems to prevent the formation of SGs. In A549 cells, RSV infection induces a redistribution of the O-linked *N*-acetylglucosamine (OGN) transferase (OGT) to IBs [58]. OGN-modification of ribosomal proteins is critical for assembly of stalled RNA and RNPs into SGs [66]. Hence, sequestration of OGT into IBs might make it unavailable for the assembly of SGs, thereby contributing to the mechanisms evolved by RSV to prevent SGs formation (Figure 2). In several virus infections, RLRs and downstream signaling molecules have been shown to localize in viral RNA-containing SGs, thereby likely facilitating a proper encounter between viral RNA and RLRs to trigger an efficient IFN antiviral response [53]. In contrast, RLRs and downstream mitochondrial antiviral-signaling protein (MAVS) adaptor were found to progress inside different populations of cytoplasmic IBs throughout the RSV replication cycle. Formation of IBs can be induced through ectopic expression of RSV N and P proteins. In this context, N interacts with MDA5 in IBs and the capacity of the cell to induce IFN production in response to Newcastle disease virus (NDV) infection is significantly decreased [67] (Figure 2). Together, these observations support the idea that RSV replication mainly takes place in IBs, but not in SGs, and that formation of IBs contributes to antagonize the IFN antiviral response (Figure 2). A major knowledge gap regarding the role of SGs and the reason why RSV has evolved various mechanisms to prevent their formation remains to be filled.

## 4. Reactive Oxygen Species (ROS) During RSV Infection: Too Much of a Good Thing

ROS produced by diverse enzymes are a double-edged sword. Low ROS levels act as redox switch for cellular signaling cascades including those involved in the regulation of the antiviral and proinflammatory response induced by paramyxoviruses [68]. In contrast, oxidative stress refers to the damaging side of excessive ROS levels. Oxidative stress, mainly associated with the oxidative burst of phagocytic cells, has long been known as an important culprit in acute and chronic lung diseases [69,70]. RNA virus-induced oxidative stress contributes to cell death, loss of immune function, increased viral replication and inflammatory response and loss of body weight, thereby contributing to pathogenesis [71]. RSV physiopathology is no exception regarding this dual role of ROS.

Antioxidant treatment significantly ameliorates RSV-induced clinical disease and pulmonary inflammation in a mouse model of infection, suggesting a causal relationship between increased ROS production and lung disease [72]. Markers of oxidative stress resulting from lipid peroxidation—malondialdehyde (MDA)—or fatty acid peroxidation—F2-8-Isoprostane—were found elevated in the nasopharyngeal secretions (NPS) of RSV-infected children [73]. Oxidative stress, measured by decreased glutathione (GSH)/oxidized glutathione (GSSG) ratio, was also measured in blood samples of RSV-infected infants exhibiting RSV-associated acute bronchiolitis [74]. Importantly, oxidative stress marker levels in the lung and blood were positively correlated with the severity of the bronchiolitis [73,74]. Although these *in vivo* observations in children are indicative of a role of RSV in the induction of oxidative stress, these data should be interpreted with caution. Indeed, use of ventilator/hyperoxia in supportive care of severe cases of bronchiolitis or infection by alternate viruses that have not been tested in patients included in the study are a potential source of oxidative stress and therefore might introduce a bias [75]. However, the direct causal link between RSV and the induction of oxidative stress is strongly underscored by studies in animal and in cell culture. The oxidative stress markers MDA and 4-hydroxynonenal (HNE) are increased in the bronchoalveolar lavages (BAL) of RSV-infected mice [72]. Increased concentrations of F2-8-Isoprostane and MDA accompanied by decreased GSH/GSSG ratio are also observed in RSV-infected A549 and small alveolar epithelial (SAE) cells, supporting a contribution of lung epithelial cells in RSV-induced oxidative stress [76,77,78].

**Figure 3:**
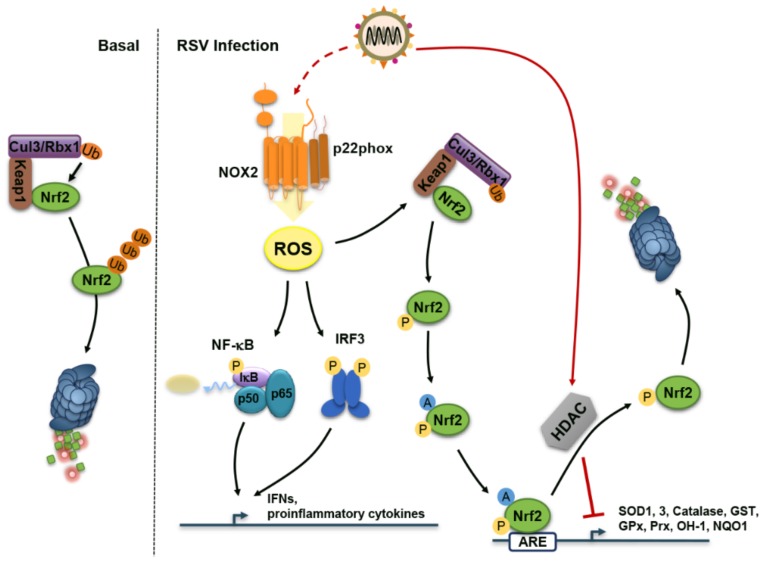
Regulation of the Oxidative Stress Response during RSV Infection. Nuclear factor erythroid 2-related factor 2 (Nrf2) is the main transcriptional activator of antioxidant genes through binding to the antioxidant responsive elements (ARE). In basal conditions, Nrf2 is sequestered in the cytoplasm by interaction with kelch-like ECH-associated protein 1 (Keap1). Keap1 also binds the E3 ubiquitin-protein ligase complex Cul3/Rbx1, which ubiquitinates Nrf2, leading to its degradation by the proteasome. RSV infection leads to production of reactive oxygen species (ROS), likely by the NADPH oxidase 2 (NOX2), which positively regulates the signaling pathways leading to expression of antiviral and proinflammatory cytokines. Additionally, ROS induce Nrf2 release from interaction with Keap1. Stabilized Nrf2 is subjected to additional regulation by phosphorylation and acetylation. RSV increases histone deacetylase (HDAC) activity leading to nuclear deacetylation of Nrf2 thereby inducing dissociation from ARE and inhibition of antioxidant genes.

Oxidative stress results from an imbalance between ROS production and detoxification by antioxidant enzymes (AOE). Oxidative stress correlates with decreased levels of AOE expression and activity at a later time of infection suggesting that oxidative damage associated with RSV infection results from an imbalance between ROS production and antioxidant cellular defenses [77]. Several reports suggest that RSV interferes with the expression of several AOE, thereby contributing to oxidative lung injury. Decreased levels of the AOE, superoxide dismutase (SOD) 1 and 3, catalase and glutathione S-transferase (GST) were observed in the NPS of children with RSV-positive bronchiolitis with the limitations of interpretation inherent to this cohort as described above [73]. Analysis of lung homogenates or BAL in a mouse model of RSV infection also showed a decline in the active levels of SOD 1-3, catalase, glutathione peroxidase (GPx), GST, peroxiredoxin (Prx) 6, heme oxygenase 1 (OH-1) and NAD(P)H:quinone oxidoreductase (NQO1) at a late time of infection [73]. Compelling evidence points to nuclear factor erythroid 2-related factor 2 (Nrf2), the main transcription factor responsible for the transcriptional regulation of AOEs through binding to antioxidant response elements (AREs), being the central target in RSV-mediated dysregulation of AOE expression [79]. Infection of Nrf2-deficient mice (Nrf2 KO) with RSV exhibits increased oxidative stress associated with elevated RSV titer and lung inflammation and injury compared to wt mice [80]. RSV infection is associated with decreased levels of nuclear Nrf2, as a result of proteasome-dependent Nrf2 degradation, ultimately leading to diminished binding to the ARE sites of AOE gene promoters [73,77,78]. Under basal conditions, Nrf2 is retained in the cytoplasm through binding to the kelch-like ECH-associated protein 1 (Keap1) inhibitor that acts as an adaptor for the Cul3/Rbx1-mediated ubiquitination and proteasome-dependent degradation of Nrf2 (Figure 3). Activation of Nrf2 upon oxidative stress conditions, results in the degradation of Keap1 and as a consequence stabilization of Nrf2. Further phosphorylation and acetylation of Nrf2 regulates nuclear translocation, DNA-binding and ARE-dependent gene transactivation [81]. Upon RSV infection of the epithelial cell lines A549 and SAE, nuclear histone deacetylase (HDAC) activity is upregulated leading to nuclear Nrf2 deacetylation and proteasome-mediated degradation (Figure 3). Blocking HDAC activity using trichostatin A (TSA) increases Nrf2 acetylation and prevents its degradation, thereby restoring RSV-induced AOE gene expression [78].

AECs organized in a pseudostratified epithelium play a primary role in the host defense against RSV infection. AECs are the first to detect RSV infection through RIG-I and MDA5 to mount an early antiviral response essentially mediated by the secretion of antiviral and proinflammatory cytokines and chemokines to limit viral replication and spreading, but also to alarm the immune system of the infection [82,83,84,85]. Use of antioxidants such as butylated hydroxyanisol (BHA) or *N*-acetylcysteine (NAC) have revealed that ROS act as a redox switch required for efficient RSV-mediated activation of the nuclear factor kappa-light-chain-enhancer of activated B cells (NF-κB), activator protein-1 (AP-1) and interferon regulatory factor (IRF) 3 activation pathways and downstream IL-8, CCL5/RANTES and CCL2/monocyte chemoattractant protein-1 (MCP-1) transcriptional expression [86,87,88,89,90,91]. Further studies showed that superoxide ions produced by the NADPH oxidase 2 (NOX2) are responsible for the redox-dependent regulation of RSV-induced NF-κB activation and downstream tumor necrosis factor alpha (TNFα) and IL-6 expression in A549 and primary normal human bronchial epithelial (NHBE) cells [92] (Figure 3). NOX2 has also been involved in the activation of IRF3-mediated antiviral response during SeV infection [93]. The observation that NOX2 acts downstream of 5’ppp-dsRNA-induced RIG-I sensing strongly suggests that this pathway is also involved in the documented redox-dependent activation of IRF3 during RSV infection [91,93] (Figure 3). The redox regulation of the IFN antiviral response during respiratory virus infection of AEC is also attributable to the dual oxidase 2 (DUOX2)-dependent H_2_O_2_ production that is required for the sustained production of IFNβ and IFNλ in AECs [94,95]. In contrast to SeV, which is a potent inducer of DUOX2, the presence of replicating RSV limits DUOX2 upregulation, suggesting that RSV has evolved strategies that target DUOX2-dependent H_2_O_2_ production to counteract the IFN-mediated antiviral response [94]. Thus, RSV faces a fine balance with ROS. While low dose of ROS is required for efficient host defense, RSV inhibition of the antioxidant response through inhibition of Nrf2 raises ROS levels to the range of oxidative stress associated with tissue damage and associated pathogenesis.

## 5. Concluding Remarks

Despite decades of research, an integrated picture of RSV-host interaction is still missing. In recent years, the first clues of how RSV modulates cellular stress responses have been unveiled. Our knowledge is still sparse and further studies are required to fully comprehend how these responses ultimately influence RSV replication and associated pathogenesis. First, the role of the ATF6 branch of the UPR and whether/how it synergizes with the antiviral function of the IRE1 pathway remains unknown. Additionally, RSV appears to have developed a very specific relationship with the SG response. Why SG formation occurs in only a subset of cells, and not others, has not yet been addressed. A detailed analysis of the kinetics of SG formation/dissociation throughout the course of RSV infection might provide important mechanistic insights into the functional interaction between SGs and RSV. In addition, several important questions need clarification: What is the role of SGs in RSV replication and/or pathogenesis? Why is a sustained SG response launched even though RSV has evolved various mechanisms to prevent it? Although not yet considered a cellular stress response, formation of cytoplasmic IBs, where replication/transcription is thought to take place, appears to be inversely correlated to SG formation. Distinct subsets of IBs are detected during the course of RSV infection. However, the characterization of these subsets is limited, the only distinction so far being size. The specific characteristics of each IB subset and whether they have any unique functions in RSV replication and pathogenesis all remain to be determined. Finally, a bimodal effect of the oxidative stress response is observed during RSV infection, with low doses being protective and higher doses contributing to pathogenesis. The identification of NOX2 and DUOX2 as sources of ROS and Nrf2 as a major culprit in the downregulation of the antioxidant defense has started to shed light on the molecular mechanisms of the redox balance. Further understanding of how levels of ROS mediate distinct functions during RSV infection will require the identification of the molecular switches targeted by ROS. Overall, although still fragmented, the current knowledge of the interaction of RSV with cellular stress responses already opens new therapeutic opportunities. These include targeting Nrf2 to restore the antioxidant response, modulating the SG/IB balance or inhibiting autophagy to relieve the negative feedback on the IRE1-dependent UPR response to restrict RSV replication.

## Abbreviations

The following abbreviations are used in this manuscript:

AEC	Airway epithelial cell
AOE	Antioxidant enzyme
AP-1	Activator protein-1
ARE	Antioxidant response element
ATF6	Activated transcription factor 6
ASK1	Apoptosis signal-regulating kinase 1
BAL	Bronchoalveolar lavage
BHA	Butylated hydroxyanisol
BiP/GRP78	Binding immunoglobulin protein
Cul3	Cullin 3
DBSA	3,5-dibromosalicylaldehide
DC	Dendritic cell
dsRNA	Double-stranded RNA
DUOX2	Dual oxidase 2
eIF	Eukaryotic translation initiation factor
ER	Endoplasmic reticulum
ERAD	ER-associated protein degradation
G3BP1	RasGAP SH3 domain-binding protein 1
GPx	Glutathione peroxidase
GSH	Glutathione
GSSG	Oxidized glutathione
GST	Glutathione S-transferase
HDAC	Histone deacetylase
HeV	Hendra virus
HN	Hemagglutinin-neuraminidase
HNE	4-hydroxynonenal
HTBE	Human tracheobronchial epithelial
IB	Inclusion body
IFN	Interferon
IRE1	Inositol-requiring enzyme 1
IRF	Interferon regulatory factor
JNK	c-Jun N-terminal kinase
Keap1	Kelch-like ECH-associated protein 1
KO	Knockout
L	Large polymerase protein
LC3b	Microtubule-associated proteins 1A/1B light chain 3B
Le	Leader
MAPK	Mitogen-activated protein kinase
MAVS	Mitochondrial antiviral-signaling Protein
MCP-1	Monocyte chemoattractant protein-1
MDA	Malondialdehyde
MDA5	Melanoma differentiation-associated protein 5
MEFs	Mouse embryonic fibroblasts
MPV	Metapneumovirus
MuV	Mumps virus
MV	Measles virus
NiV	Nipah virus
NAC	*N*-acetylcysteine
NDV	Newcastle disease virus
NF-κB	Nuclear factor kappa-light-chain-enhancer of activated B cells
NHBE	Normal human bronchial epithelial cell
NLRP3	Nucleotide-binding domain and leucine-rich repeat, pyrin domain containing 3
NOX2	NADPH oxidase 2
NPS	Nasopharyngeal Secretion
NQO1	NAD(P)H:quinone oxidoreductase
Nrf2	Nuclear factor erythroid 2-related factor 2
NS	Non-structural
OGN	O-linked *N*-acetylglucosamine
OGT	OGN transferase
OH-1	Heme oxygenase 1
PAMP	Pathogen-associated molecular pattern
PERK	PKR-like ER kinase
PIV	Parainfluenza viruses
PKR	Protein kinase R
RNP	RNA-binding protein
PRR	Pattern recognition receptor
Prx	Peroxiredoxin
Rbx1	Ring-box 1
RIDD	IRE1-dependent decay
RIG-I	Retinoic acid-inducible gene I
RLR	RIG-I-like receptor
ROS	Reactive oxygen species
RSV	Respiratory syncytial virus
SAE	Small alveolar epithelial cell
SARS-CoV	Severe acute respiratory syndrome coronavirus
SeV	Sendai virus
SG	Stress granule
SOD	Superoxide dismutase
Th2	T helper-type 2
TIA1	T-cell intracellular antigen
TIAR	TIA1-related
TLR	Toll-like receptor
TNFα	Tumor necrosis factor alpha
Tr	Trailer sequence
TRAF2	TNF receptor-associated factor 2
TrC	Complement of the Tr sequence
TSA	Trichostatin A
UPR	Unfolded-protein response
Wt	wild-type
XBP1	X-box binding protein 1
